# Activated Integrin-Linked Kinase Negatively Regulates Muscle Cell Enhancement Factor 2C in C2C12 Cells

**DOI:** 10.1155/2015/748470

**Published:** 2015-12-16

**Authors:** Zhenguo Dong, Wei Pan, Haiqing Wu, Dongjun Liu, Ming Cang

**Affiliations:** Key Laboratory of Mammalian Reproductive Biology and Biotechnology, Ministry of Education, Inner Mongolia University, Inner Mongolia, Hohhot 010021, China

## Abstract

Our previous study reported that muscle cell enhancement factor 2C (MEF2C) was fully activated after inhibition of the phosphorylation activity of integrin-linked kinase (ILK) in the skeletal muscle cells of goats. It enhanced the binding of promoter or enhancer of transcription factor related to proliferation of muscle cells and then regulated the expression of these genes. In the present investigation, we explored whether ILK activation depended on PI3K to regulate the phosphorylation and transcriptional activity of MEF2C during C2C12 cell proliferation. We inhibited PI3K activity in C2C12 with LY294002 and then found that ILK phosphorylation levels and MEF2C phosphorylation were decreased and that MCK mRNA expression was suppressed significantly. After inhibiting ILK phosphorylation activity with Cpd22 and ILK-shRNA, we found MEF2C phosphorylation activity and MCK mRNA expression were increased extremely significantly. In the presence of Cpd22, PI3K activity inhibition increased MEF2C phosphorylation and MCK mRNA expression indistinctively. We conclude that ILK negatively and independently of PI3K regulated MEF2C phosphorylation activity and MCK mRNA expression in C2C12 cells. The results provide new ideas for the study of classical signaling pathway of PI3K-ILK-related proteins and transcription factors.

## 1. Introduction

PI3K plays a key regulatory role in the proliferation and differentiation of skeletal muscle cells [1, 2]. Studies have shown that PI3K signaling is crucial for the development of muscle by directly or indirectly influencing phosphorylation of proteins such as PKB and ILK, playing an important role in proliferation, differentiation, and survival of muscle cells [3–5]. PI3K also interacts with transcription factors or proteins related to proliferation of muscle cells. Tamir and Bengal [6] found that the phosphorylation of the transcription factor MEF2C was suppressed after inhibition of PI3K activity.

Activation of ILK stimulated by growth factor is normally regulated in a PI3K-dependent manner via phosphatidylinositol (3,4,5)-trisphosphate (PIP3) interaction with the central pleckstrin homology- (PH-) like domain of ILK [7, 8]. ILK plays a very important role in myocardial regeneration [9], angiogenesis and invasion and metastasis of tumor cells [10], and matrix adhesion and signal transduction [11, 12]. Activated ILK directly affects protein phosphorylation and regulates cellular functions. ILK regulates the phosphorylation of Akt at Ser473 and of glycogen synthase kinase 3 (GSK3) in various cell types [13]. We found that ILK also directly regulates MEF2C phosphorylation in the skeletal muscle cells of goats [14].

The MEF2 proteins (MEF2A–D) consist of a family of transcriptional factors that play a central role during the development of skeletal muscle [15]. The DNA binding site of MEF2 includes a conserved DNA sequence CTA (A/T) 4TAG, present in the regulatory regions of muscle-specific genes [16, 17]. Studies have shown that the phosphorylation of the serine residue (S59) between MADS and MEF2 domain in MEF2C would enhance the DNA binding activity of MEF2C, and the residue in the MEF2 family members is conservative [18]. MEF2C interacts with the A/T enrichment region in the enhancer of the skeletal muscle-specific creatine kinase enhancer (MCK) and thus regulates and activates the expression of muscle-specific genes [19].

In summary, PI3K plays an important role in the proliferation of skeletal muscle cells. PI3K has a great impact on ILK activation. ILK also controls MEF2C-mediated regulation of skeletal muscle cell proliferation, following inhibition of phosphorylation. However, no report is available on PI3K-dependent activation of ILK directly regulating MEF2C phosphorylation during the proliferation of skeletal muscle cells. The present study explored whether activated ILK directly regulated the phosphorylation of MEF2C during the proliferation of C2C12 cells and regulates the expression of myogenic genes.

## 2. Materials and Methods

### 2.1. Materials

DMSO (Wako, KPM0962, Japan), ILK antibody (Abcam, ab15838, USA), ILK phosphorylation antibody (sc-130196), and polyclonal antibody p-MEF-2 (Ser59) (sc-13919-R) were purchased from Santa (USA); MEF2C antibody (ab64644) and *α*-tubulin antibody (ab15246) were purchased from Abcam (USA).

### 2.2. PI3K Inhibitor and ILK Inhibitor

A pharmacological inhibitor specific for the PI3K pathway, namely,* 2-(4-morpholino)-8-phenyl-4H-1-benzopyran-4-one* (LY294002), was used. Applicable cultures were supplemented with LY294002 at indicated concentrations, 30 min prior to treatment [20, 21]. A pharmacological inhibitor specific for the ILK,* N-methyl-3-(1-(4-(piperazin-1-yl)phenyl)-5-(4*′-*(trifluoromethyl)-[1,1*′-*biphenyl]-4-yl)-1H-pyrazol-3-yl)propanamide (22)* (Cpd22, Compound 22), facilitated the dephosphorylation of Akt at Ser473 and other ILK targets [22]. LY294002 was obtained from Santa Cruz Biotechnology and Cpd22 was obtained from Calbiochem, which were prepared and stored according to manufacturer's instructions.

### 2.3. Cell Culture

C2C12 cells were planted in 10 cm culture dish (1 × 10^6^ cells per dish) and maintained at 37°C and 5% CO_2_ in a humidified atmosphere and cultured in Dulbecco's Modified Eagle's Medium (DMEM) (Invitrogen) supplemented with 12% fetal bovine serum (Hyclone) and 1% penicillin/streptomycin (Sigma Aldrich).

### 2.4. MTT Cell Activity

Subsequent to different treatments, supernatants were discarded and MTT solution (5 mg MTT/mL PBS) was added to each well containing the cell monolayer and incubated for different times at 37°C in a humidified 5% CO_2_ atmosphere. After incubation, formazan crystals generated in healthy cells were dissolved in 0.6 mL of DMSO and analyzed to determine absorbance at 490 nm versus blank. Absorbance values were calculated as a percentage versus untreated controls. MTT was obtained from Sigma Aldrich (the result is in Supplementary Material available online at http://dx.doi.org/10.1155/2015/ 748470).

### 2.5. ILK RNA Interference

Three ILK short hairpin RNA (shRNA) sequences were designed as follows, with nucleotide start positions based on the NCBI entry for ILK (accession NM_001161724.1): ILK-810, 5′-GGCAGGGCAATGATATTGTTG-3′; ILK-999, 5′-GATCTCTCTACAATGTTCTAC-3′; ILK-1048, 5′-GAGCCAAGCTGTAAAGTTTGC-3′; and ILK-1763, 5′-CCCGCCTGTCACAATAAAGTT-3′. And shRNA negative control (NC-shRNA) is 5′-GTTCTCCGAACGTGTCACGT-3′. The sequences were subcloned into PGPU6/GFP/Neo vectors (GenePharma, China) and transiently transfected into C2C12 cultures.

### 2.6. Western Blot

Following treatment, cells were immediately transferred to ice and rinsed in 0.5 mL of RIPA lysis buffer (Millipore, USA). Lysates were diluted in 5 × SDS-PAGE sample loading buffer (Beijing ComWin Biotech Co., Ltd., China) and heated in a boiling water bath for 3–5 minutes in order to fully denature protein, followed by sodium dodecyl sulfate polyacrylamide gel electrophoresis (SDS-PAGE) using 12% polyacrylamide gels. Gels were run for 30 min at 90 V and 60 min at 120 V. Following SDS-PAGE, proteins were transferred to polyvinylidene fluoride (PVDF) membranes (Immobilon, Millipore, USA) using a semidry electrotransfer system (Bio-Rad, USA) for 60 min at 0.3 A (constant current). Membranes were then incubated with specific primary antibodies diluted as 1 : 500–1 : 1000 in TBS-T (1 : 1000), overnight at 4°C. The next day, membranes were incubated in anti-rabbit (or anti-goat) horseradish peroxidase-conjugated secondary antibody (Abcam, USA) for 1 h at room temperature. Antibodies were detected with the Pierce ECL Western Blotting Substrate as per the manufacturer's instructions, and bands were exposed to autoradiography film (Tanon 5200, Yuanpinghao Biotechnology Co., Ltd., China). Exposed bands were visualized and then quantified by densitometry using Image J (National Institutes of Health, USA). All bands were expressed as optical density values relative to a control on the same blot.

### 2.7. qPCR Analysis

Following treatment, the total RNA of cells was immediately extracted. Reverse transcription using 500 ng total RNA was performed with PrimeScript RT Master Mix (Takara). The MCK (GenBank Acc.: NM_007710.2) primer sequences were designed by GenePharma (China): forward primer (F): 5′-GAGGCAATATGAAGGAGGTTTTCC-3′; reverse primer (R): 5′-GGTGCTCGTTCCACATGAAGG-3′. The GAPDH (GenBank Acc.: NM 001289726.1) primer sequences were designed by Beijing Genomics Institute (BGI) (China): forward primer (F): 5′-TGTGTCCGTCGTGGATCTGA-3′; reverse primer (R): 5′-TTGCTGTTGAAGTCGCAGGAG-3′. The qPCR experiments used SYBR Premix Ex Taq (Takara) and the real-time fluorescent quantitative PCR of JENA (qTOWER 2.2, Germany). PCR conditions were 95°C for 30 s for the initial denaturation step, followed by an appropriate number of cycles at 95°C for 10 s and 60°C for 30 s. Threshold cycles (Ct) were automatically derived from the amplification plots constructed from the ROX-normalized fluorescence signals by MxPro qPCR software. Relative quantification was performed by the comparative Ct (2^−ΔΔCt^) method (*n* = 3). GAPDH was used to normalize the values.

### 2.8. Statistical Analysis

Data are presented as means ± SD (*n* = 3 for each group). The significance of differences between experimental or treated groups and control groups was determined by analysis of variance; ^*∗*^ *p* < 0.05, ^*∗∗*^ *p* < 0.01 by the *t*-test were considered statistically significant.

## 3. Results

### 3.1. Inhibition of PI3K-Dependent ILK Phosphorylation Inhibited MEF2C Phosphorylation Activity and the Expression of MCK mRNA

**Figure 1:**
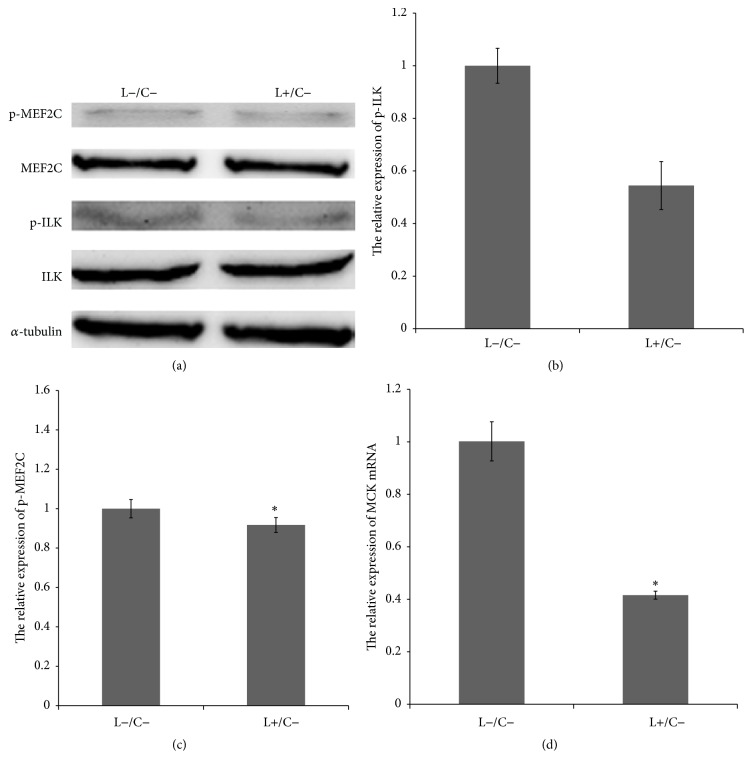
Inhibition of PI3K inhibited ILK and MEF2C phosphorylation activity and the expression of MCK mRNA. (a), (b), and (c) Changes in ILK and MEF2C expression in LY294002-treated (L+/C−) C2C12 cells were not obvious; ILK (0.544445 ± 0.091, *p* > 0.05) and MEF2C (0.9170784 ± 0.038, *p* < 0.05) phosphorylation levels decreased. The 3 h treatment with 20 *μ*M LY294002 had a clearly inhibitory effect on ILK phosphorylation and MEF2C phosphorylation levels. (d) MCK mRNA levels in C2C12 cells treated with LY294002 (L+/C−) were significantly lower (0.4156358 ± 0.015, *p* < 0.05) than that in the control (L−/C−).

We used 20 *μ*M LY294002 to treat C2C12 for 3 h to inhibit the activity of PI3K. As illustrated in Figures 1(a) and 1(b), ILK expression did not change after the inhibition of PI3K activity, but the ILK phosphorylation decreased (0.544445 ± 0.091, *p* > 0.05).

As demonstrated in Figures 1(a) and 1(c), the expression of MEF2C protein did not change after the inhibition of PI3K activity, whereas the phosphorylation level of MEF2C (S59) levels decreased significantly (0.9170784 ± 0.038, *p* < 0.05). MEF2C interacted with the enhancer region of MCK and thus enhanced the expression of MCK, and the expression of MCK was decreased significantly (0.4156358 ± 0.015, *p* < 0.05).

### 3.2. ILK Regulated MEF2C Negatively

#### 3.2.1. After the Direct Inhibition of ILK Phosphorylation, MFE2C Phosphorylation Activity and the Expression of MCK mRNA Were Significantly Increased

**Figure 2:**
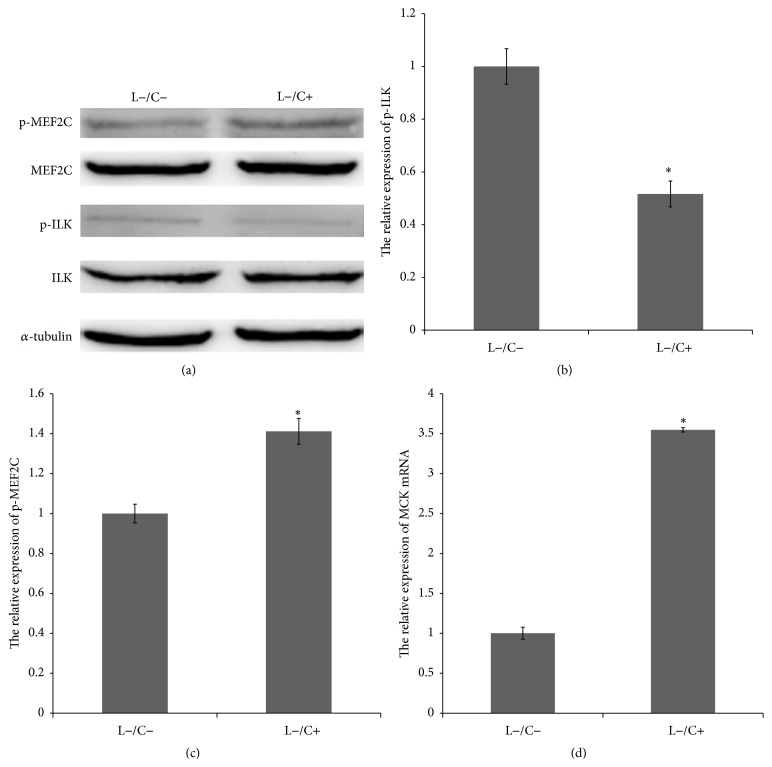
Inhibition of ILK phosphorylation activity increased MEF2C phosphorylation activity and the expression of MCK mRNA. (a), (b), and (c) Changes in ILK and MEF2C expression in Cpd22-treated (L−/C+) C2C12 cells were not obvious; ILK (0.516547 ± 0.098, *p* < 0.05) phosphorylation levels decreased significantly and MEF2C phosphorylation levels increased significantly (1.4117083 ± 0.065, *p* < 0.05). (d) MCK mRNA levels in C2C12 cells treated with Cpd22 were significantly increased (3.5478187 ± 0.027, *p* < 0.05) compared with that in the control.

After treatment of C2C12 with 10 *μ*M Cpd22, ILK expression did not change (Figures 2(a) and 2(b)), while ILK phosphorylation level significantly decreased (0.516547 ± 0.098, *p* < 0.05). As shown in Figures 2(a) and 2(c), the expression of MEF2C was not altered after the addition of ILK inhibitor Cpd22, while the phosphorylation level of MEF2C (S59) increased significantly (1.4117083 ± 0.065, *p* < 0.01) to 1.41 times that of the control group. As shown in Figure 2(d), the expression of MCK was 3.55 times that of the untreated control group, and the expression of MCK was significantly increased (3.5478187 ± 0.027, *p* < 0.05).

#### 3.2.2. ILK-shRNA Enhanced MEF2C Phosphorylation Activity and MCK mRNA Expression in C2C12

**Figure 3:**
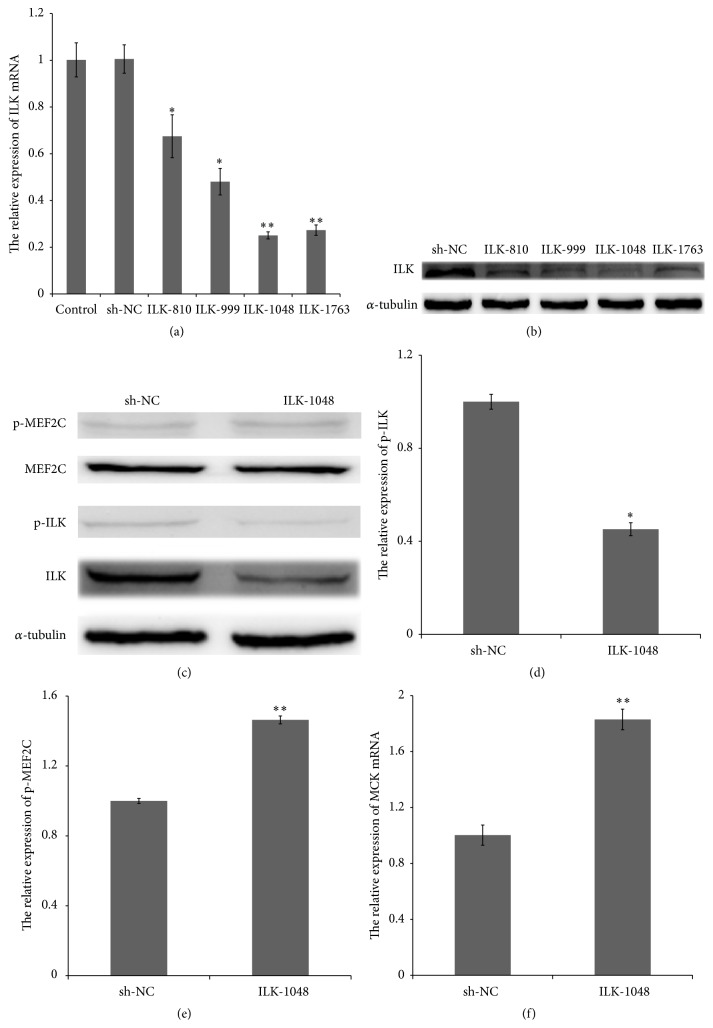
ILK-shRNA enhanced MEF2C phosphorylation activity and MCK mRNA expression. (a), (b) ILK-1048 was the most potent and selected for further study. (c), (d), and (e) Changes in MEF2C expression were not obvious with ILK-shRNA (ILK-1048) in C2C12 cells; ILK (0.45204 ± 0.027, *p* < 0.05) phosphorylation levels decreased significantly and MEF2C phosphorylation levels increased significantly (1.463772 ± 0.022, *p* < 0.01). (d) MCK mRNA levels in C2C12 cells treated with Cpd22 were significantly increased (1.829917 ± 0.070, *p* < 0.01) compared with that in the control.

After four ILK-shRNA (ILK-810, ILK-999, ILK-1048, and ILK-1763) and NC-shRNA were transfected and untransfected control cells (control) were transiently transfected into C2C12 for 48 h, control (1.00177 ± 0.073) and NC-shRNA (1.005488 ± 0.060) had similar levels of ILK mRNA (*p* > 0.05), but four ILK-shRNA had different levels of ILK mRNA (Figure 3(a)): ILK-810 (0.6749447 ± 0.0916712, *p* < 0.05); ILK-999 (0.480587304 ± 0.015, *p* < 0.05); ILK-1048 (0.2506583 ± 3.50213*E* − 05, *p* < 0.01); ILK-1763 (0.2732058 ± 0.0006739, *p* < 0.01). The similarity of the western blot results (Figure 3(b)) showed that ILK-1048 was the most potent one and was selected for further study.

We transfected C2C12 with ILK-1048 for 48 h, and then there was an obvious decrease in ILK expression and ILK phosphorylation activity (0.45204 ± 0.027, *p* < 0.05) compared with those of sh-NC (Figures 3(c) and 3(d)). However, MEF2C phosphorylation activity (1.463772 ± 0.022, *p* < 0.01) was increased distinctly (Figure 3(e)), and MCK mRNA levels (1.829917 ± 0.070, *p* < 0.01) were significantly higher than that of the control (Figure 3(f)). The similarity of the ILK-shRNA results to that of the drug treatments suggested that ILK downregulates MEF2C.

### 3.3. Inhibition of PI3K-Dependent Activation of ILK Did Not Directly and Negatively Regulate MEF2C Phosphorylation Activity and MCK Expression

**Figure 4:**
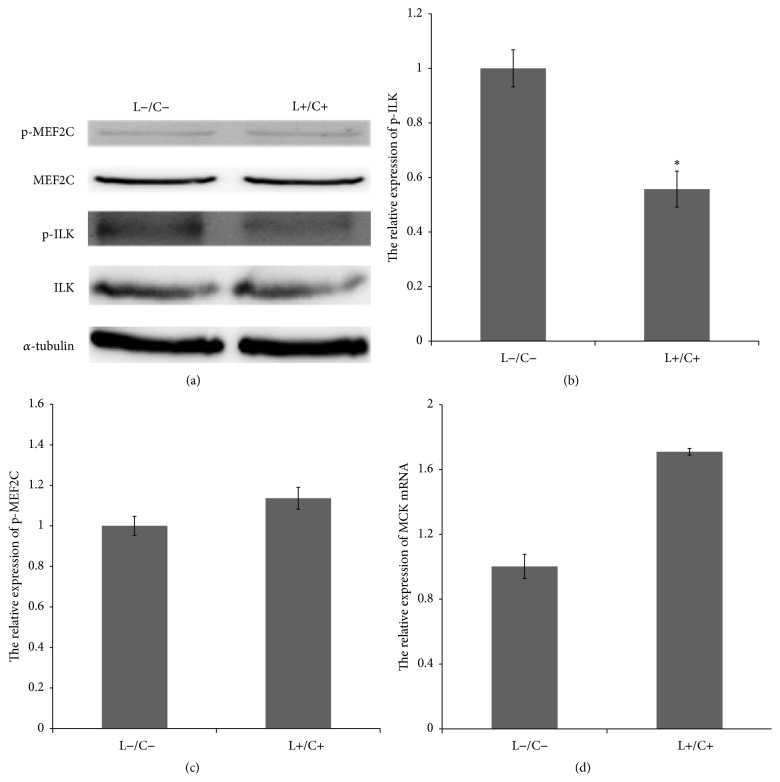
Inhibition of PI3K-dependent activation of ILK did not directly and negatively regulate MEF2C phosphorylation activity and MCK mRNA expression. (a), (b), and (c) Changes in ILK and MEF2C expression in LY294002/Cpd22-treated (L+/C+) C2C12 cells were not obvious; ILK (0.557457 ± 0.066, *p* < 0.05) phosphorylation levels decreased significantly and MEF2C phosphorylation levels increased slightly (1.1359755 ± 0.054, *p* > 0.05). (d) MCK mRNA levels in C2C12 cells treated with LY294002/Cpd22 were increased (1.7096923 ± 0.020, *p* > 0.05) compared with that in the control.

Using Cpd22, C2C12 was further treated with LY294002. As illustrated in Figures 4(a) and 4(b), ILK expression did not change, while ILK phosphorylation level was significantly decreased (0.557457 ± 0.066, *p* < 0.05).

As shown in Figures 4(a) and 4(c), the expression of MEF2C did not change after the addition of 2 inhibitors, whereas the phosphorylation level of MEF2C (S59) increased partly (1.1359755 ± 0.054, *p* > 0.05). As illustrated in Figure 4(d), the expression of MCK was 1.71 times that of control group after addition of two kinds of inhibitors (1.7096923 ± 0.020, *p* > 0.05), but it was lower than that of the experimental group following a single treatment with Cpd22 (3.55 times). Moreover, it was significantly lower than that of the experimental group following single treatment using Cpd22, which is consistent with the MEF2C protein analysis.

## 4. Discussion

The C2C12 cell line is an immortalized rat skeletal muscle satellite cell primarily isolated from the thigh bone muscle of a 2-month-old C3H female mouse after crush injury [23]. This cell line does not differentiate when cultured in the medium with 10%–20% fetal bovine serum, and it maintains the stem cell characteristics of the satellite cell, with a rapid proliferation rate. Therefore, C2C12 was selected as the skeletal muscle cell model in the present study. We found that C2C12 proliferation and growth were inhibited differently with different concentrations of LY294002 and treatment times to inhibit the activity of PI3K. With the increased drug concentration and treatment, the inhibitory role was increasingly apparent, prompting us to explore the phenomenon.

### 4.1. PI3K-Dependent ILK Activation

PI3K plays an important role in the proliferation, growth, differentiation, and apoptosis of skeletal muscle cells [12, 24]. The substrate of PI3K, phosphatidylinositol (3,4,5)-trisphosphate (PIP3) activates ILK and plays a key role in the regulation of the PKB/AKT and GSK-3 signaling pathways [25, 26]. Therefore, in the present study the ILK activity was detected by inhibiting the activity of PI3K using PI3K-specific inhibitor LY294002. We found that ILK phosphorylation was significantly decreased. The results (Figure 1) confirmed that ILK activation was PI3K-dependent, which was consistent with other reports [6, 27].

### 4.2. ILK Negatively Regulates MEF2C Phosphorylation and Transcription Activity

ILK contains three domains to support a variety of functions, including the N-terminal domain composed of four ankyrin repeats and a PH domain and the C terminal kinase domain. The unique protein structure enables ILK to play an important role in the coordination of extracellular matrix (ECM) and the signal transduction of growth factor [28, 29]. Evidence suggests that ILK may directly phosphorylate proteins associated with tumorigenesis by increasing cell proliferation, enhancing anchor independence of growth factors, and inhibition of apoptosis [14, 30, 31]. Investigations also revealed that MEF2C phosphorylation level increased significantly after ILK inhibition and knocking down, and the MCK expression that plays a vital role in the proliferation and differentiation of muscle cells increased significantly, suggesting that MEF2C transcription increased significantly after ILK inhibition or knocking down. After inhibiting ILK phosphorylation activity and mRNA expression, the phosphorylation activity of MEF2C and its transcriptional activity were significantly increased, thus indicating that ILK negatively regulates MEF2C phosphorylation and transcriptional activity.

### 4.3. PI3K Positively Regulates MEF2C Phosphorylation and Transcription

MEF2C directly regulates gene transcription of myomesin. MEF2C loss in skeletal muscle results in improper sarcomere organization. These results reveal a key role for MEF2C in maintenance of sarcomere integrity and postnatal maturation of skeletal muscle [32]. In the early stage, MEF2C phosphorylation was inhibited after PI3K inhibition [6]. Concomitantly, the expression of MCK mRNA regulated by MEF2C was significantly decreased after inhibition of PI3K activity, which was also consistent with the results of MCK protein detection after PI3K inhibition [33].

### 4.4. Inhibition of PI3K-Dependent Activation of ILK Did Not Directly and Negatively Regulate MEF2C Phosphorylation and Transcription

ILK is activated in PI3K-dependent manner and regulates the related proteins or genes in the classical signal transduction pathway. In the present study, the ILK phosphorylation was inhibited after the inhibition of PI3K activity, when both MEF2C phosphorylation and transcription exhibited significantly decreasing trends. In order to further determine the regulatory effect of ILK on MEF2C, both the phosphorylation and the transcriptional activity of MEF2C displayed significantly increasing trends after direct inhibition of ILK phosphorylation and mRNA expression. Two different experimental results explain similar inhibition of ILK phosphorylation activity. According to previous studies, ILK is directly related to MEF2C, and ILK negatively regulates the MEF2C phosphorylation and transcription activity. When two kinds of inhibitors were used, both the phosphorylation and transcription activities of MEF2C were increased, but lower than in the group treated with a single ILK inhibitor, which suggests that although the addition of PI3K inhibitor inhibited ILK activity, it did not negatively modulate MEF2C. Thus, we conclude that ILK activation independent of PI3K negatively regulates MEF2C phosphorylation and transcription in C2C12 cells.

## 5. Conclusions

As one of the key molecules linking important signaling pathways inside and outside cells, ILK plays a stellar role in the study of skeletal muscle proliferation and tumor therapy. In this study, the relationship of the PI3K/ILK/MEF2C/MCK signaling pathway with muscle proliferation was investigated using C2C12 cell line. A key finding: ILK activation was not entirely PI3K-dependent, especially in the regulation of MEF2C phosphorylation and transcription. Our next study will investigate how ILK regulates MEF2C in detail. We hope that the results of this study will provide a new impetus for the treatment of tumors and improvement of meat quality in domestic animals.

## Supplementary Material

Different concentrations of the PI3K inhibitor LY294002 were used to treat C2C12 cells for different times. With increase in the treatment time and drug concentration, the inhibition efficiency showed an increasing trend. From the result, we chose 20µM LY294002 to treat C2C12 for 3h to inhibit the activity of PI3K.

## Abbreviations

DMEM: Dulbecco's Modified Eagle's Medium
ECM: Extracellular matrix
GSK3: Glycogen synthase kinase 3
ILK: Integrin-linked kinase
LY294002: 2-(4-Morpholino)-8-phenyl-4H-1-benzopyran-4-one
MCK: Muscle-specific creatine kinase
MEF2: Myocyte enhancer factor 2
PI3K: Phosphatidylinositol 3-kinase
PKB: Protein kinase B.
