# Supplementary material for: Activated Integrin-Linked Kinase Negatively Regulates Muscle Cell Enhancement Factor 2C in C2C12 Cells
Source: Biomed Res Int. 2015 Dec 16;2015:748470. doi: 10.1155/2015/748470 (PMC4695646; doi:10.1155/2015/748470)
Supplement: Supplementary file 1 — Different concentrations of the PI3K inhibitor LY294002 were used to treat C2C12 cells for different times. With increase in the treatment time and drug concentration, the inhibition efficiency showed an increasing trend. From the result, we chose 20µM LY294002 to treat C2C12 for 3h to inhibit the activity of PI3K. [file 748470.f1.pdf]

# Supplementary materials

## The efficacy of LY294002 against PI3K activity

The concentrations of the PI3K inhibitor LY294002 concentrations used in this study were 10μM, 20μM and 30μM, respectively. The inhibitor at each concentration was used to treat-C2C12 cells for 2h, 3h and 4h.With increase in the treatment time and drug concentration, the inhibition efficiency showed an increasing trend (Fig. 1). From the result, we chose 20μM LY294002 to treat C2C12 for 3h to inhibit the activity of PI3K.

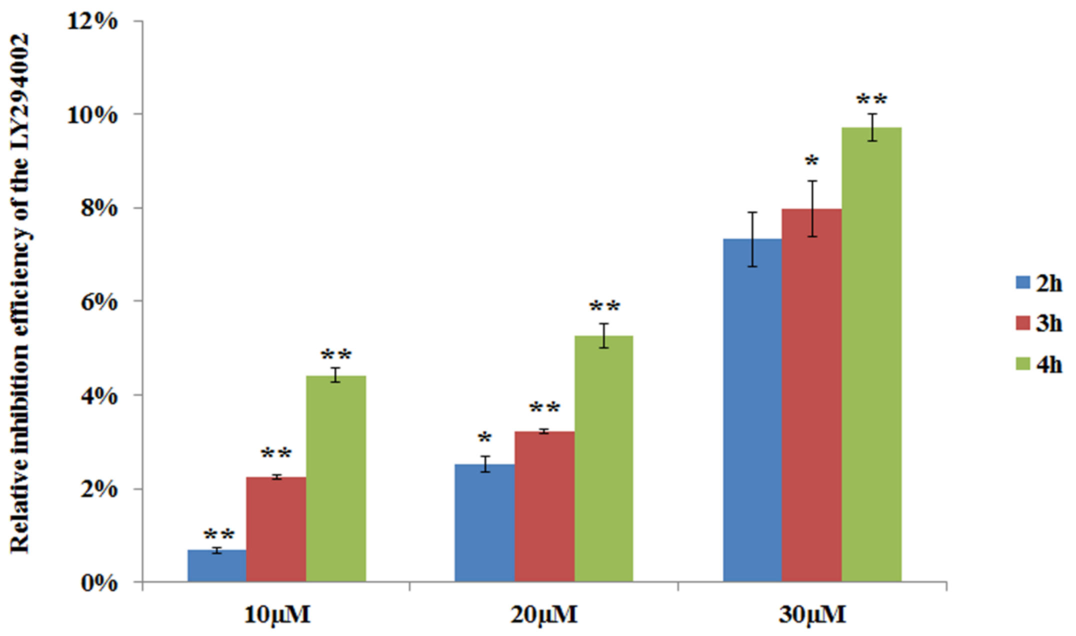


**Fig.1. The efficacy of LY294002 against PI3K activity.** C2C12 cells were treated with 10 μM/ 20 μM/30 μM LY294002 for 2h/3h/4h, and differences in their reductive capacities were measured vs. their untreated control. Results are means ± SD (n = 5 for each group). * $p < 0.05$, ** $p < 0.01$.
